# Evaluating the Accuracy of Virtual Reality in Replicating Real-Life Human Postures and Forces for Injury Risk Assessment

**DOI:** 10.3390/s24217049

**Published:** 2024-10-31

**Authors:** Xiaoxu Ji, Xin Gao, Ethan Swierski

**Affiliations:** 1 Biomedical Engineering, Gannon University, Erie, PA 16541, USA; swierski001@gannon.edu; 2 Electrical Engineering, Gannon University, Erie, PA 16541, USA; gao005@gannon.edu

**Keywords:** virtual reality, accuracy, ergonomics, evaluation, posture, spinal force

## Abstract

The objective of this study was to assess the accuracy of virtual reality (VR) technology in replicating real-life environments for the adoption of appropriate human postures and forces. Despite the widespread implementation of VR in various applications, there is a lack of research evaluating the accuracy of human postures and sensory aspects in the VR environment compared to real-life scenarios. A total of twenty-two student participants were recruited for this study, which involved a common lifting task. Two specific poses were identified as having potentially excessive forces exerted on the lower back. By comparing the angles of seven anatomical joints in both the real environment and the VR environment at each pose, we observed that depth perception may influence posture adoption in the VR setting. Moreover, the presence of a physical load applied to both hands significantly influenced the postures adopted by participants compared to those in the VR environment. These deviations in postures directly led to significant differences in predicted spinal forces exerted on the lower back, which in turn could result in inaccurate assessments of injury risks and the design of injury prevention programs. Therefore, it is crucial to understand the accuracy of VR technology as a substitute for real-life environments.

## 1. Introduction

The development of virtual reality (VR) has followed a progressive trajectory and includes contributions from multiple disciplines. It encompasses advancements in hardware, software, graphics, and content creation. VR technology enables the creation of immersive and interactive experiences, offering users a compelling sense of visual depth and perception [1], resulting in authentic and engaging virtual environments [2]. VR has found wide-ranging applications across diverse fields.

Currently, VR technology is used for valuable applications in the educational domain, enabling students to experience and visualize educational concepts instead of relying solely on mental imagery [1]. Additionally, VR technology has been employed to analyze subjects’ balance on different support surfaces [3], facilitating improved workstation design across various environments and enabling multiple perspectives in task completion [4]. Furthermore, VR has demonstrated potential in healthcare applications. Studies have shown its effectiveness in enhancing the gait of stroke patients and assessing motor function recovery exercises for upper limb and shoulder rehabilitation, such as reaching the nose with the index finger and bending the arm up to 180 degrees [5,6]. In hand surgery, VR technology has allowed for live streaming of procedures, creating a virtual reality environment for students and trainees to practice surgical techniques safely and without jeopardizing patient well-being [7]. Particularly in the context of the COVID-19 pandemic, this application has seen significant advancements, enabling practice procedures to occur in any setting rather than solely in hospitals [8]. Additionally, VR has been utilized in aviation applications, such as enhancing passenger comfort [9], assessing passengers’ seat properties [10], training aircraft attendants for routine duties, and safety [11,12,13].

The utilization of VR technology in these studies may present certain limitations, particularly in terms of depth perception, which could potentially lead to flawed results or suboptimal workstation design. The user’s field of view is restricted, contributing to inaccuracies, discomfort, or even potential falls or injuries [3]. Moreover, the use of VR technology carries potential health risks, as highlighted by LaMotte from CNN [14]. Prolonged exposure to any screen, including VR displays, may increase the risk of visual problems [15]. Thus, several studies have specifically assessed the injury risks associated with VR environments. Key areas of concern include musculoskeletal disorders [16], motion sickness [17,18], and ergonomic issues [19]. While VR technology has been employed in injury risk assessments, there is currently a lack of research analyzing the forces exerted on the body during movements [20,21], and no direct comparisons have been made to real-life forces and postures to verify the accuracy of human movement representation in VR.

Several factors may influence the discrepancies between real and virtual environments. These factors include software design, depth perception, posture, and gender-based anthropometrics. For instance, females may experience a higher likelihood of motion sickness and imbalance compared to males, who might exhibit more consistent movement across both environments [22]. Therefore, this study aimed to determine whether movements in a VR environment can accurately replicate real-life tasks, and if not, to identify the primary causes of any inaccuracies.

In the current study, a comprehensive motion analysis was conducted using VR technology in conjunction with the Xsens motion capture system and Siemens ergonomics tools to assess the full-body postures adopted by participants and the corresponding spinal forces exerted on the lower back. This analysis involved comparing the joint angles measured in the VR environment to those obtained in the real environment. The Xsens motion capture system has previously been utilized to evaluate posture in healthcare workers and brewery workers during their daily tasks, demonstrating its effectiveness in accurately analyzing worker posture and identifying high-risk tasks that may require lift assistive devices or preventive measures [23,24]. JACK Siemens software (v9.0) (Plano, TX, USA) integrates a comprehensive range of anthropometric data, human performance models, and motion prediction capabilities, making it widely adopted across diverse research areas [25,26,27,28], with its force output extensively validated. Moreover, one of the key advantages of JACK software is its integration with motion tracking systems, which significantly reduces the time required for injury assessment, thereby addressing a common limitation in traditional evaluation methods. Therefore, this study provides an opportunity to compare the postures adopted in different settings (real vs. VR). This comparative analysis aims to determine the fidelity of VR technology in representing posture and the forces exerted on the human body during various movements and environmental conditions. Considering that a small bias in posture can cause a notable difference of the force exerted on the lower back [29,30], accurate postures integrated with applied forces are crucial for robust ergonomics analyses, which makes the assessment of performance in VR essential for injury prevention.

## 2. Methods

### 2.1. Participants and Software

This study received approval from the Institutional Review Board for studies involving human participants. The sample size was determined using an a priori power calculation based on input parameters that were set for a *t*-test: one tail, an effect size of 0.6, a significance level of 0.05, and a power of 0.8. Thus, a total of twenty-two student volunteers were recruited for this study. The average body weight and body height, along with their corresponding standard deviations, are provided in Table 1. Anthropometric measurements were taken for each subject, including upper arm, lower arm, hand, thigh, shank, and foot lengths, as well as arm span, shoulder width, and hip width. These measurements were crucial for creating accurate digital human models for the study.

**Figure 1:**
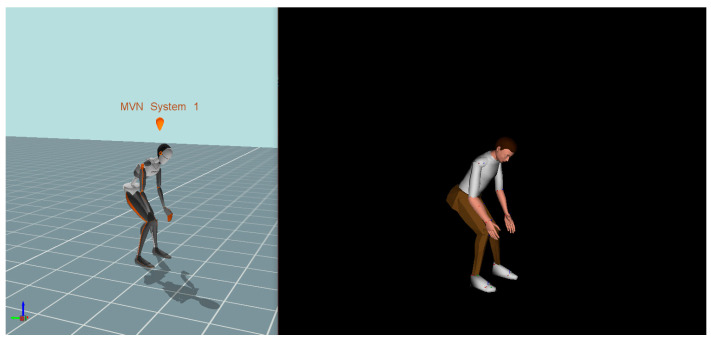
The integration of Xsens Awinda software (**Left** side) with JACK Siemens software (**Right** side). The movement of both DHMs is synchronous.

Xsens software (v2019) (Movella, Enschede, The Netherlands) [31] was used to create the first digital human model (DHM_Xsens) for recording actual human movements. A second digital human model (DHM_JACK) was generated using JACK Siemens software [32] to predict the spinal forces exerted on the participants’ lower backs. Both DHMs were created based on the anthropometric data obtained during the orientation session. In order to achieve an accurate human movement simulation, the skeletal segments of DHM_Xsens were aligned with the anatomical joint centers of DHM_JACK, leveraging the unique features of JACK. Figure 1 demonstrates the DHM_JACK on the right side, replicating the movement performed by DHM_Xsens on the left side.

### 2.2. Virtual Reality (VR) Setup

The VR environment in this study was developed using Unity 3D (Unity Technologies, San Francisco, CA, USA) [33]. The environment consisted of a table and a box, both designed using Autodesk Fusion 360 (v2020). To facilitate the use of the Oculus Rift [34] with Unity, the Oculus Integration App and the extended reality interaction toolkit were installed. The initial coordinate setup for both the VR environment and Oculus was configured to (0, 0, 0). In order to enable interaction with game objects, rigid body and collider components were added to the VR hands and VR objects. The box in the VR environment was equipped with two convex mesh colliders, positioned in the same locations as on the physical box, allowing participants to grasp it. Additionally, a rigid body component with a mass of 10 kg was assigned to the VR box.

### 2.3. Operational Tasks

**Figure 2:**
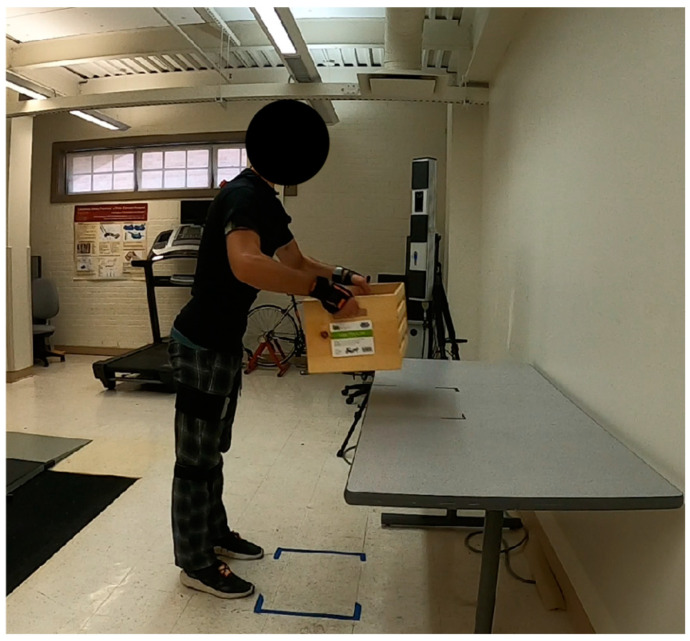
One common lifting task was designed for subjects to perform.

In this study, a common lifting task was designed, as depicted in Figure 2. Considering the safety recommendations provided by the study [35], participants were instructed to lift a 10 kg load from the floor (start position) and place it on a table (end position). The height of the table was set at 75 cm, and the anterior–posterior distance between the two positions was set to 45 cm without any medial–lateral offset. These settings were determined based on the recommended restrictions for horizontal (between 10 in and 25 in) and vertical (less than 30 in) distances for load placement [35]. Moreover, the distance between the parallel feet standing on fixed positions was set to 45 cm, corresponding to the average shoulder width [36]. To ensure consistency, the same restrictions were applied in the VR environment to maintain design fidelity for participants. In order to prevent muscular fatigue and ensure the reliability of collected data, each task was repeated four times in each of the real and VR environments.

### 2.4. Data Analysis

**Figure 3:**
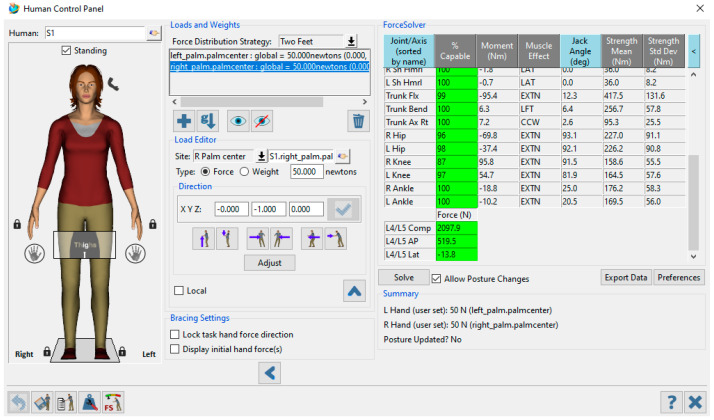
The predicted spinal forces were analyzed by using JACK Siemens software.

To mitigate anticipatory bias, each subject was instructed to perform the full motion sequence, from lifting the box off the floor to placing it on the table. Two specific postures were identified as potentially resulting in excessive forces exerted on the lower backs (specifically the 4th/5th lumbar spine) of the participants. The exerted spinal forces were influenced by the adopted postures and the loads (including the magnitude, application point, and the force direction) applied to both hands, as shown in Figure 3. For each specific posture, a comprehensive analysis was conducted to compare the spinal forces and corresponding joint angles between the two operational environments (real and VR). This analysis encompassed the assessment of the compressive force, the anterior–posterior (AP) shear force, and the angles of the trunk, hips, knees, and shoulders.

The 10 kg load was assumed to be distributed evenly on both hands. The 50 N force applied on each palm’s center was calculated by Equation (1), and the direction was vertical.

(1) $$F=\frac{m*g}{b},$$

where *m* represents the mass of the box, *g* represents the gravity acceleration, and *b* represents the number of hands.

**Figure 4:**
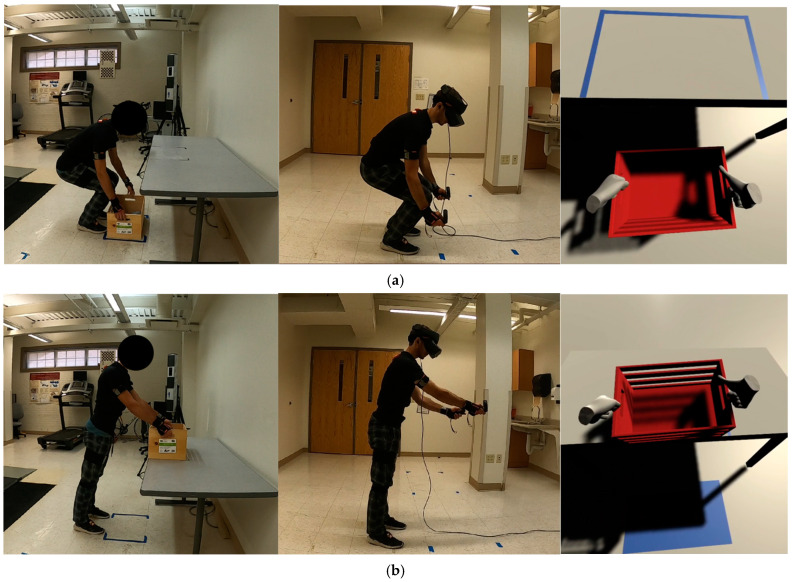
The two detected postures had excessive forces exerted on the lower backs of participants: (**a**) Pose #1—lifting the box from the floor; and (**b**) Pose #2—placing the box on the table. At each pose, the three figures from left to right represent the pose of lifting the real box, the pose of lifting the box in the VR environment, and the VR box, respectively.

Pose #1: The initial pose of interest was identified as the participants began to lift the box from the floor while in a squatting position, as depicted in Figure 4a. In this pose, the force applied to each hand was set at 50 N in a vertically upward direction.

Pose #2: The second pose of interest was identified as the moment when participants were about to place the box on the table within the designated spot, as illustrated in Figure 4b. In this pose, the force exerted on each hand remained constant at 50 N, directed upwards.

### 2.5. Statistical Analysis

A statistically significant level of 0.05 was employed in this study. For each pose, a two-way analysis of variance (ANOVA) was conducted to determine significant differences in exerted spinal forces and the aforementioned anatomical joint angles between the two operational environments (real and VR) and genders. To further comprehend the differences in postures adopted by females and males, *t*-tests were conducted to analyze the variables of spinal forces and anatomical joints. Additionally, cross-correlation (R) values were analyzed between the main anthropometric variables and the exerted spinal forces to assess the impact of these variables on the risk of injury during the common lifting task.

## 3. Results

### 3.1. Pose #1: Lifting the Box from the Floor

**Figure 5:**
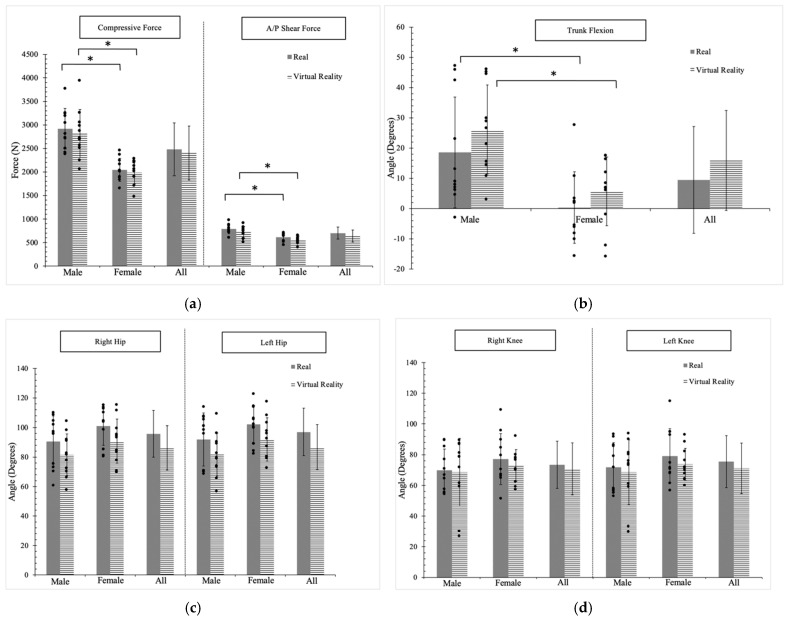

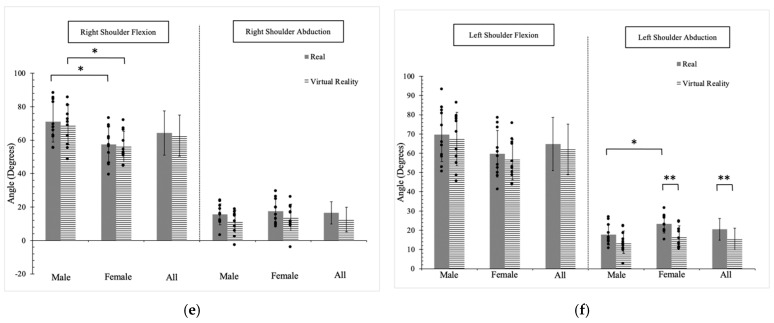
The forces exerted on the lower back and the corresponding joint angles adopted at Pose #1: (**a**) spinal forces, (**b**) trunk, (**c**) hips, (**d**) knees, (**e**) right shoulder, and (**f**) left shoulder. The positive and negative values indicate flexion/extension and abduction/adduction. * represents the significant difference between genders (males and females); ** represents the significant difference between the two environments (real and VR).

Figure 5 illustrates the plot of compressive and AP shear forces exerted on the lower back, as well as the identified anatomical joints (trunk, hips, knees, and shoulders), at this specific pose. Statistical analysis revealed significant differences (*p* < 0.05) in spinal forces between males and females in both the real environment (compressive force: males (2918.7 N), females (2045.4 N); AP shear force: males (788.7 N), females (611.1 N)) and the VR environment (compressive force: males (2816.0 N), females (1987.9 N); AP shear force: males (718.9 N), females (559.5 N)).

In the joint analysis, significant differences (*p* < 0.05) were observed between genders for trunk and right shoulder flexion. The differences in trunk flexion and right shoulder flexion between genders were approximately 20° and 14°, respectively. Although there was no significant difference in left shoulder flexion, there was still an approximate 10° difference between genders. Regarding left shoulder abduction, a significant difference was observed between genders in the real environment (*p* = 0.02), while no significant difference was found in the VR environment (*p* = 0.22). However, the difference in the joint angle ranged only from 3° to 6°, which suggests that this particular joint difference could be considered negligible. No significant differences were observed between genders or between the real and VR environments in the other anatomical joints. The specific *p* values for all comparisons are provided in Table 2, Table 3 and Table 4.

Table 5 and Table 6 present the correlation coefficient (R) values. Table 5 focuses on the correlation between trunk angles and other variables. At Pose #1, trunk angles exhibited a high correlation with compressive spinal forces in both the real (R = 0.72) and VR (R = 0.69) environments. Moreover, trunk angles demonstrated a strong correlation with shoulder flexion, with R values of 0.69 in the real environment and 0.70 in the VR environment for right shoulder flexion and 0.66 in the real environment and 0.72 in the VR environment for left shoulder flexion. Notably, trunk angles displayed a negative correlation with hip flexion, with R values exceeding 0.80. Table 6 provides the correlation between hip angles and other variables. At this specific pose, the hip angles demonstrate a moderate correlation with knee flexion and shoulder movements, with approximate R values of 0.50.

### 3.2. Pose #2: Placing the Box on the Table

**Figure 6:**
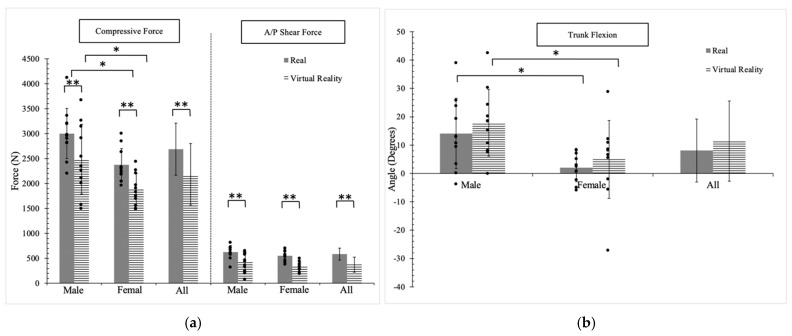

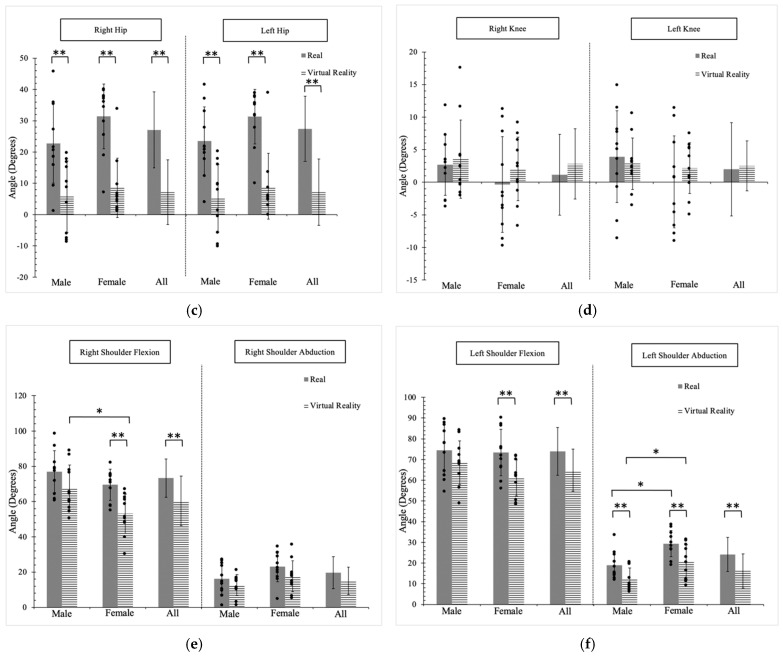
(**a**) The forces exerted on the lower back and the corresponding joint angles adopted at Pose #2: (**a**) spinal forces, (**b**) trunk, (**c**) hips, (**d**) knees, (**e**) right shoulder, and (**f**) left shoulder. The positive and negative values indicate flexion/extension and abduction/adduction. * represents the significant difference between genders (males and females); ** represents the significant difference between the two environments (real and VR).

Figure 6 illustrates the plot of exerted spinal forces and the corresponding adopted anatomical joints at Pose #2. The exerted compressive force showed significant differences between the real (2688.1 N) and VR (2185.1 N) environments. Additionally, significant differences were observed between genders in both the real environment (male: 3002.1 N, female: 2374.2 N) and the VR environment (male: 2491.1 N, female: 1879.1 N). While there was a significant difference in the exerted AP shear force between the two environments, the *p* values were greater than 0.05 for the comparison between genders.

In the joint analysis, significant differences were observed between the real and VR environments for all anatomical joints except the trunk, right shoulder abduction, and knees. Notably, the hips and shoulders exhibited larger angular movements in the real environment compared to the VR environment. The differences in hip angles reached approximately 20°, while the differences in shoulder angles reached approximately 10°.

When comparing males and females, significant differences were observed in trunk flexion/extension angles in both the real environment (*p* = 0.008) and VR environment (*p* = 0.03). Furthermore, left shoulder abduction exhibited significant differences in both environments (real: *p* = 0.001; VR: *p* = 0.01). Additionally, significant differences in right shoulder flexion were observed only in the VR environment (*p* = 0.01). Detailed *p* values can be found in Table 7, Table 8 and Table 9.

Table 10 presents the correlations of trunk angles with other variables. At Pose #2, trunk angles continued to exhibit a correlation with the exerted compressive spinal forces in both the real (*p* = 0.63) and VR (*p* = 0.55) environments. Additionally, trunk angles displayed a negative correlation with hip flexion, with R values of approximately −0.74 in the real environment and −0.54 in the VR environment.

Table 11 provides the correlations of hip angles with other variables. The hip angles demonstrate a relative correlation with knee flexion, specifically in the real environment, with R values ranging from approximately 0.56 to 0.63.

## 4. Discussion

This study aimed to assess the accuracy of postures adopted in a VR environment with the physical movements performed in the real environment. Specifically, the participants were tasked with lifting a weight from the floor and placing it on a table. We evaluated the positions of seven anatomical joints (trunk, hips, knees, and shoulders) and examined the resulting spinal forces, including compressive and AP shear forces, exerted on the lower back during this task.

At Pose #1, there were significant differences in the adopted postures between genders, which aligns with the findings in [37,38]. It was observed that due to variations in biomechanics between genders, females exhibited a higher degree of hip flexion while maintaining a more neutral trunk position when lifting the box from the floor [37,38]. Conversely, male participants showed a preference for greater trunk flexion rather than hip flexion compared to females. This led to a notable discrepancy in compressive force, with males experiencing over 800 N more force, and in AP shear force, with males exerting over 150 N more force on their lower backs than females in both the VR and real environments. These discrepancies can be attributed to the fact that extensive trunk flexion may increase compressive forces on spinal discs [39,40]. Furthermore, the statistical analysis revealed a strong correlation between trunk flexion and compressive force, which supports the findings in [23].

At Pose #1, a significant negative correlation was observed between the trunk and hips. Since the positions of the boxes were fixed during the experiment, participants had to flex their hips and knees while keeping their trunks in a neutral position in order to reach the objects. Our statistical analysis revealed a correlation ranging from 0.617 to 0.755 between the hips and knees, providing a clear explanation for the greater flexion of the hips and knees observed in females compared to males. Additionally, trunk flexion showed a correlation with shoulder flexion. As the trunk flexed forward, the shoulders exhibited a corresponding increase in flexion to effectively pick up objects. Consequently, males displayed more than 10° of increased shoulder flexion compared to females at Pose #1. Although there was a significant difference in left shoulder abduction between the VR and real environments (*p* = 0.003), the observed 5° difference could be attributed to the variation in standing positions and was thus considered negligible.

At Pose #1, although there were significant differences in certain joint angles between genders that resulted in variations in exerted spinal forces, no differences were observed when comparing the two environments. This can be attributed to the fact that all participants exhibited a large range of motion that included the trunk, hips, and knees in order to successfully complete the lifting task from the floor at this specific pose. Given the substantial range of motion, small variations in joint angles between environments were not considered statistically significant. Furthermore, the weight of the object played a significant role in the lack of differences between the two environments, as no actual physical load was exerted by the participants’ hands at Pose #1.

At Pose #2, significant differences in trunk flexion and exerted compressive spinal force persisted when comparing genders, primarily due to the considerable trunk flexion exhibited by males. The correlation between these variables further supports the findings in [23,38]. Additionally, there were notable disparities in shoulder abduction between genders. Since the dimensions of the object were fixed and considering that the average shoulder width is generally larger in males compared to females [36], females exhibited relatively smaller shoulder abduction. These observed gender-specific characteristics in the lifting task were consistent at both Pose #1 and Pose #2.

However, when comparing the two environments at Pose #2, significant differences were observed in the exerted spinal forces and nearly all corresponding joint angles, except for the knees and trunk. While the difference in trunk angles was not pronounced, there was an approximate 20° difference in hip angles between the two environments. This suggests that hip flexion plays a crucial role in influencing the exerted spinal forces, aligning with the findings in our previous study [23].

Furthermore, the average flexion/extension and abduction/adduction angles of both shoulders were greater in the real environment compared to the VR environment. The discrepancy in shoulder angles can be attributed to the adopted postures of the hips and trunk. In the VR environment, where participants had a small hip flexion angle (approximately 7°) and maintained a neutral trunk position (approximately 3°), participants exhibited a corresponding small shoulder flexion and abduction to place the object on the table. Conversely, in the real environment, participants displayed a larger hip flexion angle (approximately 27°) and a similar trunk flexion angle, resulting in a greater shoulder flexion and abduction required to physically place the object. These deviations in hip and shoulder angles may be a result of visual errors experienced in the VR environment, leading participants to mistakenly believe that the object had been successfully placed on the table. However, the primary factor contributing to the significant differences in postures at Pose #2 could also be attributed to the weight of the object. Despite efforts to replicate the real lifting task by setting the object’s mass in the VR environment, the sensory experience in VR does not fully replicate the feeling of the real environment. Consequently, the reaction forces transmitted to both hands are noticeably different. When lifting a heavier object, participants tend to bring it closer to the upper body to maintain a balanced center of gravity [41]. This explanation effectively accounts for the observed larger shoulder abduction in the real environment compared to the VR environment.

During the analysis of R values at Pose #2, a high correlation was observed between trunk angles and compressive force, while a negative correlation was found between trunk and hip angles. These correlations were consistent with those observed at Pose #1. Furthermore, in the real environment, hip flexion exhibited a correlation with shoulder flexion, whereas this correlation was absent in the VR environment due to the absence of hip flexion. Additionally, there was a correlation between hip and knee angles at both poses when the positions of the objects were fixed in the experiment.

Based on the evaluation conducted in this study, it has been observed that depth perception in the VR environment can lead to deviations in motion. Depth perception plays a crucial role in posture adoption within VR environments, as the brain relies on integrating sensory inputs like vision, proprioception, and balance to guide movement and maintain stability [42,43]. Adapting to altered visual cues or changes in depth perception [44,45] requires the brain to recalibrate, which may result in postural adjustments to preserve balance, spatial orientation, and effective interaction with the virtual environment. Particularly in cases where the range of motion is relatively small, such as at Pose #2, the impact of visual errors on posture adoption is more pronounced compared to Pose #1, where the range of motion is larger. However, the load applied to both hands emerges as a significant factor that greatly influences the postures adopted by participants. These differences in postures directly affect the corresponding exerted spinal forces. In the real environment, the compressive and AP shear forces were measured at 2688.1 N and 588.3 N, respectively. However, in the VR environment, these forces exerted on the lower back were measured at 2185.1 N and 375.1 N. It is worth noting that the weight of the object used in this study was 10 kg. If the weight of the object were to increase, the compressive and AP shear forces would also increase, potentially surpassing the recommended safety thresholds of 3400 N [46] and 700 N [47]. However, due to the inaccuracies in the adopted postures, the estimated spinal forces in the VR environment are lower than these safety thresholds. These estimated errors can lead to erroneous evaluations of the risk of injury, placing participants in potential danger in real-life situations.

## 5. Conclusions

This study aimed to analyze the accuracy of postures during a common lifting task in the VR environment compared to the real environment. Two specific poses were examined, representing scenarios where maximum spinal forces were exerted on the lower backs of participants. Through this analysis, notable differences in postures were observed between genders, which directly influenced the exerted spinal forces. It is important to acknowledge that while visual errors may contribute to variations in adopted postures between the two environments, the impact of object weight on injury assessment must be taken into account in the VR environment. In light of these findings, it is crucial for engineers to prioritize the design of a VR environment that can accurately replicate sensory experiences for injury assessment and prevention.

## Figures and Tables

**Table 1:** The participants’ body weight and body height (mean ± standard deviation).

Subjects	Number	Body Mass (kg)	Body Height (cm)
Males	11	78.1 ± 5.7	179.6 ± 3.4
Females	11	67.2 ± 7.9	164.6 ± 4.2

**Table 2:** All the statistical *p* values compared between the real and VR environments at Pose #1 are listed. P1 is used to represent Pose #1; Trunk_Flex represents trunk flexion and extension; R_Hip represents right hip flexion/extension; L_Hip represents left hip flexion/extension; R_Sh_Flex represents right shoulder flexion/extension; R_Sh_Abd represents right shoulder abduction/adduction; L_Sh_Flex represents left shoulder flexion/extension; L_Sh_Abd represents left shoulder abduction/adduction; R_Knee represents right knee flexion/extension; and L_Knee represents left knee flexion/extension.

P1	Compressive	A/P Shear	Trunk_Flex	R_Hip	L_Hip	R_Sh_Flex	R_Sh_Abd	L_Sh_Flex	L_Sh_Abd	R_Knee	L_Knee
Real	2482.1	699.9	9.4	95.8	97.0	64.3	16.6	64.8	20.5	73.5	75.5
VR	2402.0	639.2	15.9	86.1	86.8	62.7	12.6	62.0	15.4	70.8	71.1
*p* values	0.48	0.06	0.14	0.96	0.05	0.63	0.07	0.48	0.003	0.6	0.4

**Table 3:** The *p* values between males and females in the real environment at Pose #1.

P1_Real	Compressive	A/P Shear	Trunk_Flex	R_Hip	L_Hip	R_Sh_Flex	R_Sh_Abd	L_Sh_Flex	L_Sh_Abd	R_Knee	L_Knee
Males	2918.7	788.7	18.5	90.5	91.9	71.1	15.6	69.7	17.7	69.8	71.8
Females	2045.4	611.1	0.3	101.0	102.1	57.5	17.5	59.8	23.3	77.1	79.2
*p* values	1 × 10^−5^	2 × 10^−4^	0.01	0.12	0.14	0.01	0.52	0.09	0.02	0.28	0.32

**Table 4:** The *p* values between males and females in the VR environment at Pose #1.

P1_VR	Compressive	A/P Shear	Trunk_Flex	R_Hip	L_Hip	R_Sh_Flex	R_Sh_Abd	L_Sh_Flex	L_Sh_Abd	R_Knee	L_Knee
Males	2816.0	718.9	26.1	81.5	81.7	69.2	11.3	67.4	13.9	68.6	68.5
Females	1987.9	559.5	5.7	101.0	102.1	56.2	13.8	56.7	16.9	73.0	73.7
*p* values	1 × 10^−4^	1 × 10^−3^	2 × 10^−3^	0.62	0.11	9 × 10^−3^	0.45	0.05	0.22	0.55	0.47

**Table 5:** The cross-correlation values between the trunk and other variables at Pose #1 are listed. P1 is used to represent Pose #1; Comp represents the exerted compressive force; AP represents the exerted AP shear force; Trunk (Real) represents the adopted trunk angles in the real environment; and Trunk (VR) represents the adopted trunk angles in the VR environment.

P1	Comp	A/P	R_Hip	L_Hip	R_Sh_F	R_Sh_A	L_Sh_F	L_Sh_A	R_Knee	L_Knee
Trunk (Real)	0.72	0.41	−0.86	−0.83	0.69	−0.36	0.66	0.16	−0.24	−0.28
Trunk (VR)	0.69	0.49	−0.41	−0.80	0.70	−0.39	0.72	0.27	−0.25	0.22

**Table 6:** The cross-correlation values between the hip and other variables at Pose #1 are listed. R_Hip (Real) represents the adopted right hip angles in the real environment; L_Hip (Real) represents the adopted left hip angles in the real environment; R_Hip (VR) represents the adopted right hip angles in the VR environment; and L_Hip (VR) represents the adopted left hip angles in the VR environment.

P1	Comp	A/P	Trunk	R_Sh_F	R_Sh_A	L_Sh_F	L_Sh_A	R_Knee	L_Knee
R_Hip (Real)	−0.45	−0.09	−0.86	−0.50	0.40	−0.48	−0.30	0.43	0.52
L_Hip (Real)	−0.43	−0.13	−0.83	−0.41	0.51	−0.37	−0.31	0.50	0.62
R_Hip (VR)	−0.22	−0.15	−0.41	−0.49	0.40	−0.38	−0.64	0.52	0.51
L_Hip (VR)	−0.33	−0.15	−0.80	−0.37	0.41	−0.28	−0.58	0.50	0.54

**Table 7:** All the statistical *p* values compared between the real and VR environments at Pose #2 are listed. P2 is used to represent Pose #2.

P2	Compressive	A/P Shear	Trunk_Flex	R_Hip	L_Hip	R_Sh_Flex	R_Sh_Abd	L_Sh_Flex	L_Sh_Abd	R_Knee	L_Knee
Real	2688.1	588.3	8.1	27.1	27.4	73.3	19.6	74.0	24.1	1.2	2.0
VR	2185.1	375.1	11.4	7.2	7.2	60.4	15.1	64.8	16.2	2.8	2.5
*p* values	1 × 10^−3^	4.4 × 10^−6^	0.33	5 × 10^−7^	8 × 10^−8^	5 × 10^−4^	0.07	8 × 10^−3^	4 × 10^−4^	0.36	0.77

**Table 8:** The *p* values between males and females in the real environment at Pose #2.

P2_Real	Compressive	A/P Shear	Trunk_Flex	R_Hip	L_Hip	R_Sh_Flex	R_Sh_Abd	L_Sh_Flex	L_Sh_Abd	R_Knee	L_Knee
Males	3002.1	626.3	14.1	22.8	23.5	77.0	16.2	74.6	18.9	2.7	3.9
Females	2374.2	550.3	2.1	31.4	31.3	69.6	23.1	73.4	29.4	−0.4	0.1
*p* values	2 × 10^−3^	0.14	8 × 10^−3^	0.1	0.08	0.11	0.08	0.82	1 × 10^−3^	0.26	0.21

**Table 9:** The *p* values between males and females in the VR environment at Pose #2.

P2_VR	Compressive	A/P Shear	Trunk_Flex	R_Hip	L_Hip	R_Sh_Flex	R_Sh_Abd	L_Sh_Flex	L_Sh_Abd	R_Knee	L_Knee
Males	2491.1	420.0	17.8	5.8	5.3	67.7	12.6	68.5	12.0	3.6	2.8
Females	1879.1	330.2	4.9	8.6	9.0	53.1	17.6	61.2	20.4	2.1	2.2
*p* values	0.02	0.17	0.03	0.54	0.41	0.01	0.13	0.09	0.01	0.53	0.69

**Table 10:** The cross-correlation values between the trunk and other variables at Pose #2 are listed. P2 is used to represent Pose #2; Comp represents the exerted compressive force; AP represents the exerted AP shear force; Trunk (Real) represents the adopted trunk angles in the real environment; and Trunk (VR) represents the adopted trunk angles in the VR environment.

P2	Comp	A/P	R_Hip	L_Hip	R_Sh_F	R_Sh_A	L_Sh_F	L_Sh_A	R_Knee	L_Knee
Trunk (Real)	0.63	0.17	−0.74	−0.74	0.11	−0.20	0.10	0.07	−0.34	−0.30
Trunk (VR)	0.55	0.35	−0.52	−0.54	0.31	0.12	0.28	0.21	−0.38	−0.36

**Table 11:** The cross-correlation values between the hip and other variables at Pose #2 are listed. R_Hip (Real) represents the adopted right hip angles in the real environment; L_Hip (Real) represents the adopted left hip angles in the real environment; R_Hip (VR) represents the adopted right hip angles in the VR environment; and L_Hip (VR) represents the adopted left hip angles in the VR environment.

P2	Comp	A/P	Trunk	R_Sh_F	R_Sh_A	L_Sh_F	L_Sh_A	R_Knee	L_Knee
R_Hip (Real)	−0.09	0.41	−0.74	0.38	0.04	0.38	−0.12	0.56	0.56
L_Hip (Real)	−0.10	0.41	−0.74	0.42	0.06	0.44	−0.13	0.56	0.63
R_Hip (VR)	0.12	0.42	−0.52	−0.02	−0.27	0.08	−0.06	0.36	0.20
L_Hip (VR)	0.16	0.44	−0.54	−0.02	−0.27	0.06	−0.02	0.28	0.31

## Data Availability

Data is contained within the article.
